# Quantitative Chemical Profiling of Commercial Glyceride Excipients *via*^**1**^H NMR Spectroscopy

**DOI:** 10.1208/s12249-020-01883-x

**Published:** 2020-12-03

**Authors:** Isha Saraf, Varun Kushwah, Hansjoerg Weber, Dattatray Modhave, Thean Yeoh, Amrit Paudel

**Affiliations:** 1 Research Center Pharmaceutical Engineering (RCPE) GmbH, Inffeldgasse 13, 8010 Graz, Austria; 2 Institute of Organic Chemistry, Graz University of Technology, Stremayrgasse 9A, 8010 Graz, Austria; 3 Drug Product Design, Pharmatherapeutics Pharmaceutical Sciences, Worldwide Research and Development, Pfizer Inc, Groton, Connecticut USA; 4 Institute for Process and Particle Engineering, Graz University of Technology, Inffeldgasse 13, 8010 Graz, Austria

**Keywords:** quantitative proton nuclear magnetic resonance (qHNMR), isomers, method development, monoglyceride, diglyceride, triglyceride

## Abstract

Glycerides are the main components of oils, and fats, used in formulated products in the food and cosmetic industry as well as in the pharmaceutical product industry. However, there is limited literature available on the analysis of the chemical composition of glycerides. The lack of a suitable analytical method for complete chemical profiling of glycerides is one of the bottlenecks in understanding and controlling the change in chemical composition during processing, formulation, and storage. Thus, the aim of the present study is to develop a calibration-free quantitative proton nuclear magnetic resonance (qHNMR) method for the simultaneous quantification of different components of glycerides. The qHNMR method was developed for the quantification of mono-, di-, and triglycerides; their positional isomers; free fatty acids; and glycerol content. The accuracy, precision, and robustness of the developed method were evaluated and were found suitable for the quantitative analysis of five batches of marketed excipient. The study demonstrates the potential of qHNMR method for the quantification of different components of glycerides in various marketed products. The method has the ability to identify the variability of glycerides among different batches and suppliers in terms of chemical composition and also to discern the changes during storage.

**Supplementary Information:**

The online version contains supplementary material available at 10.1208/s12249-020-01883-x.

## INTRODUCTION

Glycerides are the main component of oils, emulsifiers, and fats and are extensively used in food, cosmetics, lubricants, and pharmaceutical products. Chemically, they contain mono-, di-, and triglycerides that are synthesized *via* esterification of glycerol molecules and fatty acid chains. In spite of being a common ingredient of diverse products, the exact composition and quantitative profile of glycerides are rarely reported (1,2). To date, different components of glycerides (mono-, di-, and triglycerides; glycerol; and fatty acids) are quantified using chromatography with diverse detectors. These methodologies are time-consuming, which usually necessitate complex sample preparation procedures (*e.g.*, derivatization) and calibration steps with internal or external reference standards. The method recommended in the compendial monograph for these ingredients includes wet assays and colorimetry that are either qualitative or semi-quantitative, to the best (3).

**Fig. 1:**
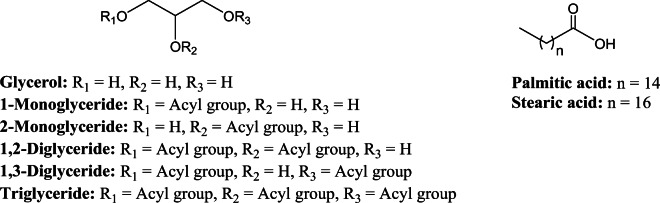
Structure of glyceride contents of mono/diglycerides (MDGs)

On the basis of the position of ester linkage, each of mono- and diglycerides exists in two positional isomeric forms, *i.e.*, 1-monoglycerides (1-MG) and 2-monoglycerides (2-MG), 1,2-diglycerides (1,2-DG) and 1,3-diglycerides (1,3-DG), respectively. Including triglycerides (TG), glycerides contain five components of glycerides, free fatty acid (FA), and glycerol (G) (Fig. 1). Previous studies have used qHNMR in characterizing and quantifying the different hydrolytic products of triglycerides during lipolysis (4,5). However, the accuracy, precision, and robustness of such methods have not been well-documented, and at the same time, simultaneous quantification of FA and G along with the positional isomers of MG and DG has not been reported.

Crystalline solid states of glycerides are well-known to exhibit the metastable α and the stable β polymorphism. The occurrence of one or both of these solid forms of glyceride and the extent and kinetics of polymorphic transformation are reported to depend upon the chemical composition (6,7). The consequence of such solid form diversity and phase transition can be morphological changes like lipid blooming and the loss of encapsulation efficiency, stabilization, and release of actives of the formulated systems containing these glycerides. In this way, the assessment of precise quantitative profiles of components in glyceride is crucial for predicting and controlling the quality of the product and ensuring the targeted shelf life. Therefore, a reliable analytical method for the quantification of different constituents of glyceride including the isomers is important. In this study, we have selected mono/diglycerides (MDGs) as a model excipient for quantitative method development. MDGs are widely used stabilizer in products such as ice creams, cakes, mayonnaise, and peanut butter (8). In addition, MDGs are extensively used in the pharmaceutical industry as emulsifiers, solubilizers, emollients, and stabilizers (9,10). Due to its wide application, several reports are available regarding the presence of α and β polymorphs of the MDGs resulting in altered water holding capacity (11,12). However, limited or no literature is available, demonstrating the effect of aging on the chemical composition of the MDGs (including isomers). In continuation of the present work, we also aim to establish correlating the current findings with the solid-state transformation of the MDG as a result of aging.

Quantitative NMR spectroscopy (qHNMR) is a precise, accurate, and calibration-free method used for the quantification of active pharmaceutical ingredients (API) and excipients (13). The qHNMR method has many advantages compared to the other chromatographic methods, especially for the excipient analysis. For example, once the method is developed, the sample analysis is fast, which requires a simple experimental procedure. The qHNMR can be used to distinguish and quantify the positional isomers of glycerides. In recent times, qHNMR has received attention for the analysis of complex samples of natural origin and food materials.

In the present work, we developed and qualified a quantitative solution-state ^1^H NMR (qHNMR) for the quantification of different possible components of the MDG such as mono-, di-, and triglycerides and positional isomers of C16 and/or C18 saturated fatty acids (1-MG, 2-MG, 1,3-DG, 1,2-DG, and TG), glycerol (G), and free fatty acids (FA) (Fig. 1). The change in chemical composition can affect the critical physico-chemical properties of the excipients Therefore, the change in different components of the excipient as a function of aging was also investigated using the developed method. Unique non-coinciding proton NMR signals of each 1-MG, 2-MG, 1,2-DG, and TG were used to quantify them by the developed method, whereas NMR signals from 1,3-DG, glycerol, and FA are in the regions corresponding to other isomers. Therefore, an indirect approach was implemented for the quantification of the latter. This way, entire chemical composition of glycerides was quantitatively profiled.

## MATERIALS AND EQUIPMENT

### Standards and Mixtures

For the method development, standard compounds, such as 1-monopalmitin, 2-monopalmitin, 1,2-dipalmitin, 1,3-dipalmitin, tripalmitin, glycerol, and palmitic acid (Sigma Aldrich, Vienna, Austria), were used. 1,2,4,5-Tetrachloro-3-nitrobenzene (tCNB) (Sigma Aldrich, Vienna, Austria) was used as a reference standard for qHNMR analysis. For the qualification, different mixtures (mixtures 1, 2, and 3) of the abovementioned standard compounds were prepared in predefined ratios and were analyzed using the developed qHNMR method, whereas the method verification was performed using five different lots (B1 to B5) of Geleol™ (Mono and Diglycerides NF from Gattefosse). Deuterated chloroform and deuterated water (Eurisotop, France) were used as solvents. All the chemicals used were of analytical reagent grade.

### Equipment

The ^1^H NMR spectra were recorded on a Varian/Agilent INOVA 500 spectrometer operating at 499.84 MHz with a 5-mm indirect detection probe. Data processing was carried out with VnmrJ 2.2D software.

## METHOD DEVELOPMENT

### Sample Preparation

Approximately 5.0 mg of pure standard compounds and 10.0 mg of MDG samples were weighed in a 2.0-mL microcentrifuge tube (Eppendorf, Vienna, Austria). Thereafter, 600 μL of deuterated chloroform, containing 1.0 mg of the reference standard (tCNB; ^1^H NMR signal at 7.76 ppm non-overlapping with glycerides, G, and FA), was added into the microcentrifuge tube. Approx. 50 μL of deuterated water (D_2_O) was added to the samples to exchange the hydroxyl protons of G. The samples were then dissolved using vortex agitation for 60 s. The tubes were centrifuged at 5000 rpm for 5 min at room temperature for the complete mixing of the samples. The solution was transferred into NMR tubes (Eurisotop, France), and the ^1^H NMR spectra were acquired.

### Quantitative Proton NMR Method

Initial experiments were conducted to identify the suitable instrumental parameters (range of relaxation delays and recycling times) required to obtain accurate quantitative results. After optimization, the acquisition parameters selected were as follows: spectral width 7997.6 Hz, relaxation delay 1.0 s, number of scans 32, acquisition time 2.049 s, and pulse width 45°. The spectra were baseline corrected, and the chemical shift of the spectra was calibrated using the chemical shift of the reference standard (tCNB). Each spectrum was recorded in triplicate, and data provided are average values with the standard deviations.

The quantity of the analyte (glycerides with different degree and sequence of substitution, glycerol, and FA) in the studied samples was determined using the following equation:

1 $$ Wx=\frac{Ax}{As}\times \frac{Ns}{Nx}\times \frac{Mx}{Ms}\times Ws $$

where

*Wx*: is the weight of the analyte (per 0.6 mL of the solution),
*Ws*: is the weight of standard,
*Ax*: is the value of integral for analyte,
*As*: is the value of integral of standard,
*Nx*: is the number of protons of analyte integrated,
*Ns*: is the number of protons of standard integrated,
*Mx*: is the molecular weight of analyte,
*Ms*: is the molecular weight of standard.

## METHOD QUALIFICATION

### Qualification Parameters

The developed method for the simultaneous quantification of various components of glycerides, free G, and FA was qualified using fit-for-the-purpose parameters such as recovery (accuracy), repeatability (intra-day precision), system suitability, intermediate precision, and robustness. The main aim of the method qualification (a non-extensive form of validation) was to verify the usefulness of the method and to demonstrate the reproducibility of the obtained results.

The acceptance criterion for each parameter was appropriately set based on the general validation requirement, the experience, and the relevant literature. Furthermore, in a pharmaceutical context, the lipid components under study are pharmacologically non-active ingredients and are classified as pharmaceutical excipients (14–19).

The method qualification was performed using standard compounds (glycerol, mono- and diglycerides, their isomers, triglycerides, and fatty acids) individually and different mixtures (mixtures 1, 2, and 3; of the known concentration of standard compounds) in triplicate. The samples (individual and mixtures) were dissolved in 0.6 mL of CDCl_3_ containing 1.0 mg of the reference standard (tCNB) as per the protocol mentioned in the “Sample Preparation” section. Initially, the system suitability of the developed method was evaluated. Briefly, the samples were analyzed six times (*n* = 6) and were evaluated for the change in chemical shift value. Thereafter, the method was qualified by evaluating recovery using the formula (Recovery (%) = (100 × amount found/original amount spiked)).

The samples were scanned six times independently to assess the repeatability (intra-day precision). Furthermore, the same sample set was also analyzed on different days to evaluate intermediate precision (inter-day precision). The qHNMR acquisition parameters, such as the number of scans (16, 32, and 64 scans) and NMR relaxation times (1 s, 15 s, and 20 s), were also varied in order to evaluate the method robustness.

## METHOD VERIFICATION USING COMMERCIAL SAMPLES

In order to verify the method, the components (different glycerides, glycerol, and fatty acids) of the five different commercial MDG (Geleol™) batches (B1 to B5) were analyzed using the established qHNMR method. These batches were obtained from Gattefosse (Saint-Priest, France) (Table I). Prior to analysis, the samples were kept at ambient temperature and humidity in closed containers. There was no additional control in place regarding the number of times each container had been opened to the environment for sampling purposes.

**Table I:** Batches of Geleol™, Mono-diglycerides (MDGs) Analyzed in This Study with Corresponding Manufacturing Date and Approximate Age

Study batch number	Geleol™, mono-diglycerides (MDGs)	
	Manufacturing date	Approximate age at time of analysis
B1	November 2015	39 months
B2	November 2016	27 months
B3	December 2017	13 months
B4	March 2018	10 months
B5	May 2018	8 months

## RESULTS AND DISCUSSION

### Method Development

**Fig. 2:**
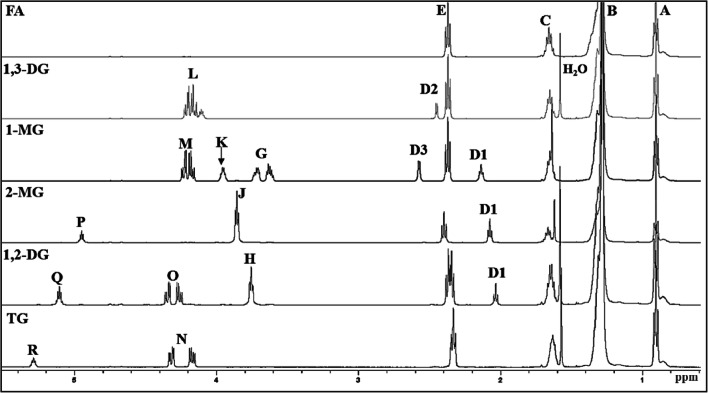
^1^H NMR spectra in CDCl_3_ of standard compounds: tripalmitin (TG), 1,2-dipalmitin (1,2-DG), 2-monopalmitin (2-MG), 1-monopalmitin (1-MG), 1,3-dipalmitin (1,3-DG), and palmitic acid (FA). The signal letters are identical as in Table II

Figure 2 shows the spectral assignment of the protons of the different standard compounds such as 1-MG, 2-MG, 1,2-DG, 1,3-DG, TG, G, and FA, and the values are listed in Table II. tCNB was selected as the reference standard as its signal (7.76 ppm) was non-overlapping with signals of glycerol and esters. The percentages of the different components of the glycerides were calculated by using Eq. 1, in which the molecular weight of analyte (*Mx*) was calculated as an average molecular weight of palmitic acid and stearic acid or their respective glycerol esters. Moreover, the areas of different spectral signals (*Ax*) and the number of protons that generated the signal (*Nx*) in Eq. 1 were obtained from the NMR spectra (Fig. 2). The components having specific non-overlapping signals in the spectrum, namely 1-MG, 2-MG, 1,2-DG, and TG, were determined by using the following equations:

2 $$ {A}_{1-\mathrm{MG}}={A}_{\mathrm{K}}\ \left({N}_{1-\mathrm{MG}}\right) $$

3 $$ {A}_{2-\mathrm{MG}}={A}_{\mathrm{P}}\ \left({N}_{2-\mathrm{MG}}\right) $$

4 $$ {A}_{1,2-\mathrm{DG}}={A}_{\mathrm{Q}}\ \left({N}_{1,2-\mathrm{DG}}\right) $$

5 $$ {A}_{TG}={A}_{\mathrm{R}}\ \left({N}_{\mathrm{TG}}\right) $$

where *N*_1-MG_, *N*_2−MG,_ *N*_1,2−DG_, and *N*_TG_ are equal to 1.

**Table II:** ^1^H NMR Signals in CDCl_3_ of the Main Protons of Glycerol; Mono-, Di-, and Triglycerides; and Fatty Acids Present in Glyceride

Signal	Chemical shift (ppm)	Multiplicity	Functional group	
			Type of proton	Compound
A	0.88–0.92	t	– C**H**_**3**_	Acyl groups and FA
B	1.22–1.37	m	– (C**H**_**2**_)_n_ –	Acyl groups and FA
C	1.60–1.70	m	– OCO – CH_2_ – C**H**_**2**_ –	Acyl groups and FA
			– COOH – CH_2_ – C**H**_**2**_ –	
D1	2.05–2.12	s	– CHO**H**, – CH_2_O**H**	Hydroxyl groups in MG and DG
D2	2.47–2.50	s		
D3	2.51–2.55	s		
E	2.28–2.36	dt	– OCO – C**H**_**2**_ –	Acyl groups in TG
	2.30–2.36	m	– OCO – C**H**_**2**_ –	Acyl groups in 1,2-DG
	2.35–2.39	t	– OCO – C**H**_**2**_ –, – COOH – C**H**_**2**_ –	Acyl groups in 1-MG, 1,3-DG, and FA
	2.38–2.43	t	– OCO – C**H**_**2**_ –	Acyl groups in 2-MG
F	3.55–3.66	m	HOC**H**_**2**_ – CH(OH) – C**H**_**2**_OH	Glycerol
G	3.60–3.75	m	ROCH_2_ – CH(OR) – C**H**_**2**_OH	Glyceryl group in 1-MG
H	3.72–3.75	m	ROCH_2_ – CH(OR) – C**H**_**2**_OH	Glyceryl group in 1,2-DG
I	3.76–3.81	dd, dd	HOCH_2_ – C**H**(OH) – CH_2_OH	Glycerol
J	3.82–3.88	t	HOC**H**_**2**_ – CH(OR) – C**H**_**2**_OH	Glyceryl group in 2-MG
K	3.92–3.98	m	ROCH_2_ – C**H**(OR) – CH_2_OH	Glyceryl group in 1-MG
L	4.05–4.24	m	ROC**H**_**2**_ – C**H**(OH) – C**H**_**2**_OR	Glyceryl group in 1,3-DG
M	4.14–4.26	ddd	ROC**H**_**2**_ – CH(OR) – CH_2_OH	Glyceryl group in 1-MG
N	4.14–4.35	dd, dd	ROC**H**_**2**_ – CH(OR) – C**H**_**2**_OR	Glyceryl group in TG
O	4.23–4.38	ddd	ROC**H**_**2**_ – CH(OR) – CH_2_OH	Glyceryl group in 1,2-DG
P	4.93–4.97	m	HOCH_2_ – C**H**(OR) – CH_2_OH	Glyceryl group in 2-MG
Q	5.08–5.13	m	ROCH_2_ – C**H**(OR) – CH_2_OH	Glyceryl group in 1,2-DG
R	5.26–5.31	m	ROCH_2_ – C**H**(OR) – CH_2_OR	Glyceryl group in TG

The signal letters agree with those given in Fig. 2

*Abbreviations*: *t* triplet, *m* multiplet, *s* singlet, *dt* doublet of triplets, *dd* doublet of doublets, *ddd* doublet of doublets of doublets

*A*_K_, *A*_P_, *A*_Q_, and *A*_R_ are the area of the corresponding signals indicated in Fig. 2 and Table II.

The determination of the 1,3-DG requires consideration that there is an overlapping between signal L and signals M, N, and O of 1-MG, TG, and 1,2-DG respectively. Thus, the area of signal L was determined by the following equation:

6 $$ {A}_{1,3-\mathrm{DG}}={A}_{4.05-4.38}-2{A}_{1-\mathrm{MG}}-2{A}_{1,2-\mathrm{DG}}-4{A}_{\mathrm{TG}}\ \left({N}_{1,3-\mathrm{DG}}\right) $$

where *A*_4.05–4.38_ represents the area of the spectrum signals L, M, N, and O, comprised between 4.05 and 4.38 ppm, and *N*_1,3-DG_ is equal to signals from five protons.

Likewise, signals representing glyceryl backbone (signals F and I) are overlapping with signals G, H, and J of 1-MG, 1,2-DG, and 2-MG respectively. Thus, the area of free glycerol signals was calculated using the following equation:

7 $$ {A}_{\mathrm{Glycerol}}={A}_{3.55-3.88}-2{A}_{1-\mathrm{MG}}-4{A}_{2-\mathrm{MG}}-2{A}_{1,2-\mathrm{DG}}\ \left({N}_G\right) $$

where *A*_3.55–3.88_ represents the area of the spectrum signals F, G, H, I, and J, comprised between 3.55 and 3.88 ppm, and *N*_*G*_ is equal to signals from five protons.

FA was calculated from the area of the signal E representing the protons on carbon atoms in alpha position with respect to the carbonyl and carboxyl groups of acyl chains and FA, respectively. Thus, the FA content was determined by using this equation:

8 $$ {A}_{\mathrm{FA}}={A}_{2.28-2.43}-2{A}_{1-\mathrm{MG}}-2{A}_{2-\mathrm{MG}}-4{A}_{1,2-\mathrm{DG}}-4{A}_{1,3-\mathrm{DG}}/5-6{A}_{\mathrm{TG}}\ \left({N}_{FA}\right) $$

where *A*_2.28–2.43_ is the area of the spectrum signal E, comprised between 2.28 and 2.43 ppm, and *N*_*FA*_ is equal to signals from owing to two protons.

### Method Qualification

**Fig. 3:**
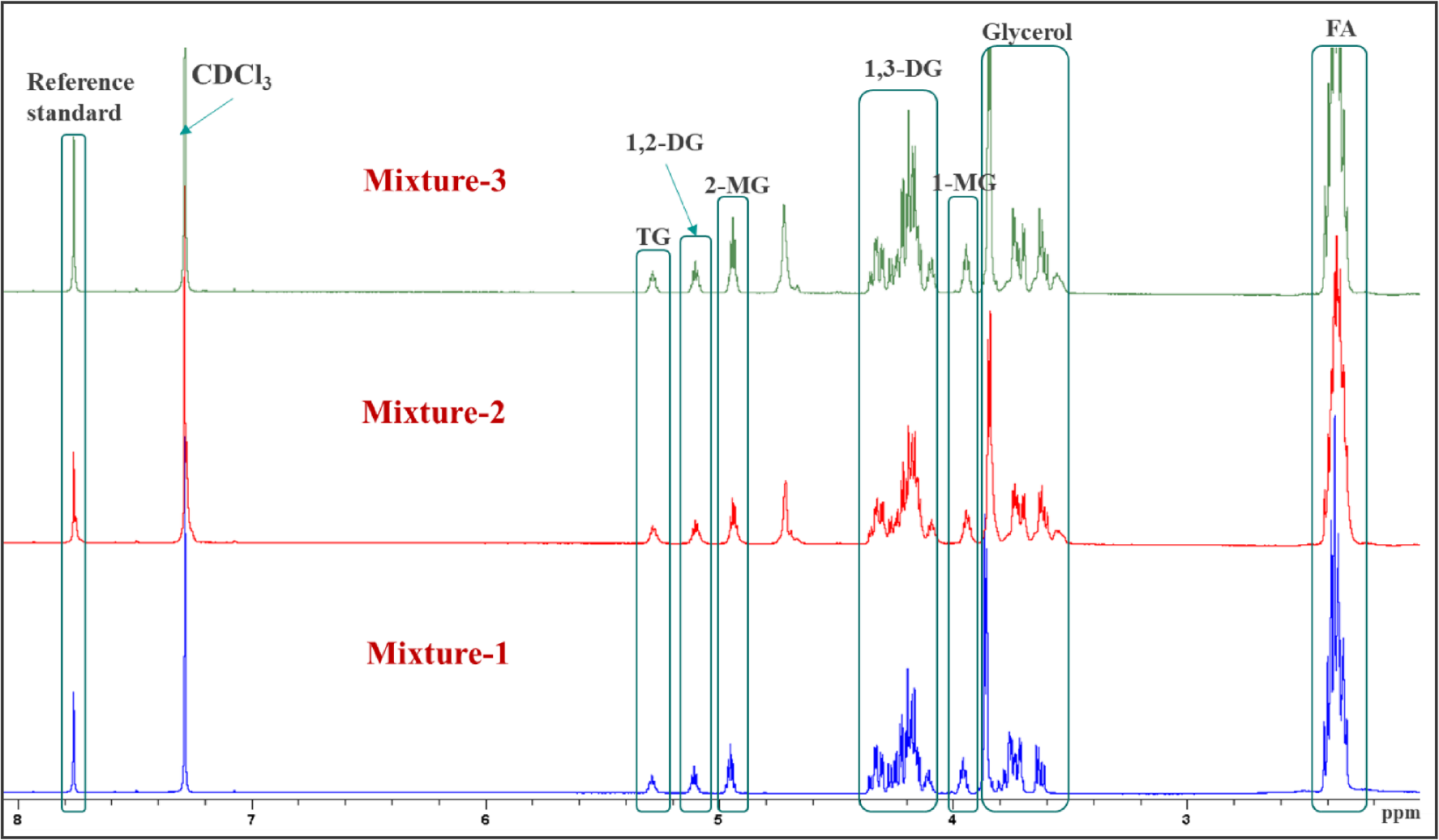
^1^H NMR spectra (in CDCl_3_) of standard mixtures (mixture 1, mixture 2, and mixture 3) containing 1-monopalmitin (1-MG), 2-monopalmitin (2-MG), 1,2-dipalmitin (1,2-DG), 1,3-dipalmitin (1,3-DG), tripalmitin (TG), glycerol, and palmitic acid (FA), together with the highlighted regions selected for the qHNMR

Figure 3 depicts NMR spectra, of mixture standard samples, comprising regions selected for the quantification of different glycerides, glycerol, and fatty acid. The ^1^H NMR spectrum of blank (containing 0.6 mL of the solvent only) showed two signals, one singlet at *δ* 7.26 for the solvent residual signal of CDCl_3_ and the other singlet set at *δ* 0.00 for the internal standard tetramethyl silane (TMS) (data not shown). No other signals were observed in the region used for quantitation. In the case of samples, one singlet at *δ* 7.76 corresponding to one aromatic proton was observed in the NMR spectrum of the reference standard (tCNB). The signals used for quantification of 1-MG, 2-MG, 1,2-DG, and TG were very well-resolved from other signals present in the mixture and the reference standard (Fig. 3), whereas NMR signals of 1,3-DG, glycerol, and FA were found to be in the regions corresponding to other glycerides. Thus, the area of signals used for quantification of 1,3 DG, glycerol, and fatty acid was determined using Eqs. 6, 7, and 8, respectively.

#### System Suitability Test (SST)

For all the test runs, the chemical shift values of the standard solutions were found to be less than the acceptance criteria of 0.2 ppm. The insignificant change in the chemical shift values demonstrated the suitability (resolution and reproducibility) of analysis (instrument, sample, and analysts) for the quantification of lipid components (13,20–23).

#### Recovery (Accuracy)

The recovery studies (accuracy) were carried out (*n* = 3), and the percentage recovery was calculated. As evident from Table III, approx. 1.2 mg of the standard samples was mixed and quantified using the developed method. The recovery of all the components of the mixture was found to be within the acceptance criterion of 95.0–105.0 %. Thus, the developed method for the quantification of different components of the glycerides was found to be accurate.

**Table III:** Results of Recovery Studies from qHNMR Analysis

Sample name	Amount (in mg)	1-MG	2-MG	1,2-DG	1,3-DG	TG	Glycerol	FA
Mixture 1	Amount of analyte in mixture	1.26	1.26	1.22	1.22	1.34	1.25	1.27
	Amount found	1.32	1.31	1.17	1.26	1.31	1.30	1.33
	Recovery %	104.71	104.12	95.86	103.23	97.90	103.87	105.07
Mixture 2	Amount of analyte in mixture	1.23	1.29	1.22	1.23	1.35	1.25	1.33
	Amount found	1.25	1.29	1.17	1.26	1.30	1.26	1.33
	Recovery %	101.84	99.87	96.22	102.42	96.50	100.67	100.37
Mixture 3	Amount of analyte in mixture	1.20	1.39	1.18	1.24	1.14	1.25	1.17
	Amount found	1.15	1.44	1.14	1.30	1.16	1.23	1.21
	Recovery %	95.93	103.64	97.01	104.97	102.11	98.27	103.45

#### Repeatability (Intra-day Precision)

The standard mixture sample containing approx. 1.2 mg of each individual component of the glyceride was quantified six times using the developed qHNMR method for the analysis repeatability (intra-day precision) study (13,21,22). As evident from Table IV, the % RSD values of the amount of all individual components were found to be in accordance with the acceptance criteria of ≤ 5.0% RSD. Thus, the developed qHNMR method was found to possess intra-day precision.

**Table IV:** Results of Repeatability Studies (Intra-day Precision) for qHNMR Analysis

	Sr. no.	1-MG	2-MG	1,2-DG	1,3-DG	TG	Glycerol	FA
Amount found (in mg)	1	1.32	1.31	1.17	1.26	1.31	1.30	1.33
	2	1.39	1.29	1.15	1.28	1.30	1.40	1.25
	3	1.38	1.30	1.15	1.29	1.30	1.37	1.30
	4	1.35	1.26	1.11	1.25	1.24	1.40	1.30
	5	1.25	1.26	1.11	1.19	1.25	1.39	1.36
	6	1.25	1.27	1.09	1.24	1.24	1.40	1.38
Average		1.32	1.28	1.13	1.25	1.27	1.38	1.32
SD		0.07	0.02	0.03	0.03	0.03	0.04	0.05
% RSD		4.95	1.71	2.70	2.70	2.51	2.94	3.67

#### Intermediate Precision (Inter-day Precision)

To determine the intermediate precision (inter-day precision), the same sample was analyzed on two different days (day 0 and day 1). As evident from Table V, a systemic decrease in the quantity of the tri-, di-, and monoglycerides was found in the case of day 1 analysis as compared to day 0. The decrease in the quantity of glycerides could possibly be due to the hydrolysis of glycerides to yield glycerol and fatty acids upon the storage of the solution. Thus, for the inter-day precision, two consecutive days were selected for the quantification of the components in order to avoid false-negative results.

**Table V:** Results of Intermediate Precision (Inter-day Precision) for qHNMR Analysis

	Sr. no.	1-MG	2-MG	1,2-DG	1,3-DG	TG	Glycerol	FA
Amount found (*n* = 6) (in mg)	Day 0	1.32	1.28	1.13	1.25	1.27	1.38	1.32
	Day 1	1.26	1.22	1.10	1.20	1.25	1.39	1.38
Individual SD (day 0)		0.07	0.02	0.03	0.03	0.03	0.04	0.05
Individual SD (day 1)		0.04	0.06	0.05	0.04	0.05	0.06	0.03
Individual % RSD (day 0)		4.95	1.56	2.65	2.40	2.36	2.90	3.79
Individual % RSD (day 1)		3.17	4.92	4.55	3.33	4.00	4.32	2.17
Average (day 0 and day 1)		1.29	1.25	1.12	1.23	1.26	1.39	1.35
Pooled SD		0.06	0.04	0.04	0.04	0.04	0.05	0.04
Pooled % RSD		4.42	3.58	3.70	2.89	3.27	3.68	3.05

Thereafter, the inter-day precision was evaluated demonstrating pooled % RSD to be in agreement with the acceptance criteria of ≤ 5.0% RSD in case of quantification of all the components of the mixture (Table V) (13). Thus, the developed method was found to be inter-day precise and accurate.

#### Solution Stability

The solution of all components was stable up to 48 h when stored in refrigerated conditions (2–8°C). Furthermore, the recovery of all the components in the solution was found to be within the acceptance criterion of 95.0–105.0%.

#### Robustness

The robustness of the method was evaluated by deliberately changing the two parameters, *i.e.*, number of scans and relaxation time. The number of scans was varied from 16 to 64, whereas the relaxation time was changed from 5 to 20 s. As evident from Table VI, the percentage assay of all components of the mixture was found to be within the limit of 90.0 – 110.0 % w/w for both the parameters. The method is capable of quantifying glyceride components with the accuracy of ± 10%, upon extreme changes of the selected parameters. In addition, the chemical shift values were found to be within the limit of ± 0.2 ppm for each set of parameters. The insignificant change in the results demonstrates the robustness of the developed qHNMR method.

**Table VI:** Results of Robustness Studies for qHNMR Analysis

Parameters		% w/w						
		1-MG	2-MG	1,2-DG	1,3-DG	TG	Glycerol	FA
Number of scans	16	96.97	95.42	94.02	100.79	96.18	106.15	92.48
	64	98.48	96.18	96.58	102.38	96.95	107.69	92.48
Relaxation time	15 s	93.18	96.95	100.00	96.03	94.66	96.92	103.76
	20 s	90.15	95.42	98.29	92.06	92.37	94.62	105.26
Change in chemical shift	< 0.2 ppm							

### Method Verification Using Commercial Samples

Table VII shows the results obtained using the qHNMR, the percentages of the 1-MG, 2-MG, 1,2-DG, 1,3-DG, TG, glycerol, and FA in five commercial MDG batches. The composition of five different batches of commercial MDGs was found to evidently alter with storage time. The quantity of 1-MG, glycerol, and free fatty acid was found to be higher in the case of aged samples, whereas the quantity of 2-MG and 1,2-DG was found to be lower in case of the aged samples as compared to the fresh samples. No marked difference in the TG and 1,3-DG was observed. This result verifies that the qHNMR method has sufficient sensitivity and selectivity to be able to discriminate between different batches of commercially available MDGs. The increase in glycerol and free fatty acid component could be due to hydrolysis of the 1-MG, 2-MG, 1,2-DG, 1,3-DG, and TG during storage. Till date, no investigation is reported to understand the change in isomer composition of MDGs with aging. Thus, a thorough assessment of change in chemical composition and its correlation with the physical structures of the MDGs is required to be further explored. The chemical profiling of different components of MDGs *via* qHNMR can aid in the in-depth mechanistic evaluation of the polymorphic transformation of MDGs and other glycerides as a function of the batch to batch variation and storage condition.

**Table VII:** Percentages (%) of the Different Glycerides, Glycerol, and Fatty Acids Present in Five Different Batches of MDG Determined by ^1^H NMR

MDG lots	Percentage (w/w) ± SD (*n* = 3)						
	1-MG	2-MG	1,2-DG	1,3-DG	TG	Glycerol	FA
B1	64.30 ± 0.33	0.84 ± 0.05	1.91 ± 0.05	17.59 ± 0.55	8.47 ± 0.48	1.62 ± 0.15	5.28 ± 0.22
B2	61.53 ± 0.36	1.22 ± 0.05	5.09 ± 0.16	15.27 ± 0.24	11.12 ± 0.37	1.22 ± 0.07	4.10 ± 0.20
B3	56.95 ± 0.43	1.21 ± 0.06	5.25 ± 0.25	21.17 ± 0.63	11.45 ± 0.44	1.43 ± 0.07	2.66 ± 0.28
B4	49.93 ± 0.33	2.23 ± 0.11	10.83 ± 0.13	19.97 ± 0.56	12.98 ± 0.39	1.20 ± 0.12	2.80 ± 0.12
B5	52.37 ± 0.29	1.75 ± 0.09	10.09 ± 0.52	19.67 ± 0.09	10.99 ± 0.28	2.11 ± 0.17	2.40 ± 0.23

## PRACTICAL RELEVANCE OF THE METHOD

For several decades, the excipient industries (including lipid surfactant manufacturer) still largely rely on nonspecific and conventional techniques such as chromatographic separation for the quantitative analysis of different components of lipids. The chromatographic separation methods mainly performed using the size exclusion chromatography (SEC). During the chromatographic separations, the low and high molecular weight components demonstrated high and low retention time in the porous column, resulting in the quantification of different components of the lipids. However, the current practiced method, due to similar molecular weight, is incompetent for the determination of different levels of isomers present in the samples. This results in the broad set of specifications of chemical composition and other associated quality attributes of the excipient, especially when the latter is the mixture of several chemical components. In this context, the qHNMR method presented herein can act as a versatile and QC-friendly method for the spectral fingerprinting and quantitative estimation of the various components of triglycerides. As a primary and absolute method, qHNMR spectroscopy is a well-accepted analytical technique in the pharmaceutical regulatory context. We demonstrated it here using the MDG as an example. The application of the current method is certainly not only limited to the pharmaceutical grades of lipid surfactant excipient but can also be extended to the food and cosmetic industry. The selectivity of the method towards all the components including positional isomers enables monitoring/improving the excipient quality during manufacturing (thus minimize lot-to-lot variability). Moreover, the qHNMR method can be applied for assuring the storage stability of the glycerides. The qHNMR analytical approach for glycerides can be further extended to complex lipid derivatives that are used as functional ingredients in the formulated products. Especially, different well-known 2D NMR spectroscopic methods such as COSY, HMBC, and HMQC can be exploited for establishing a quantitative analytical method for complex mixtures and formulations presenting overlapping peaks. Furthermore, the use of chemometric methods and other multivariate data analysis framework will be useful for the application of qHNMR method to the complex glyceride formulations (24). Here, we presented qualification following typical ICH recommendation to the chromatographic method. The inclusion of qHNMR in future method validation recommendation by ICH, which is currently under revision, will expand the method’s utility in a broader context (25–27).

## CONCLUSION

In this work, the quantitative ^1^H NMR method was developed rendering the simultaneous analysis of glycerides and positional isomers and related components including glycerol and free fatty acids for a commonly known function ingredient, MDG. The method was appropriately qualified to ensure analytical reliability during the quantification of multiple chemical components of MDG. The developed calibration-free method was found to be accurate, precise, and robust. The proposed method can be beneficial and convenient over the other existing analytical methods in terms of simplicity, accuracy, and selectivity towards the positional isomers. The application of the present qHNMR can be extended to analogous glycerides and derivatives.

## Supplementary Information

The supporting information contains T_1_ relaxation time experiments.

ESM 1 (DOCX 564 kb)

## References

[1] Barison A, Da Silva CWP, Campos FR, Simonelli F, Lenz CA, Ferreira AG (2010). A simplemethodology for the determination of fatty acid composition in edible oils through ^1^H NMR spectroscopy. Magn Reson Chem.

[2] Guillén MD, Ruiz A (2003). Rapid simultaneous determination by proton NMR of unsaturation and composition of acyl groups in vegetable oils. Eur J Lipid Sci Technol.

[3] USP-NF Mono- and Di-glycerides. Available from: https://online.uspnf.com/uspnf/document/1_GUID-EDFBCC40-7304-4BAE-9D27-239F4A6FA6F5_3_en-US?source=QuickSearch&highlight=NFMonographs%3A Mono- and Di-glycerides. Accessed 2020 Oct 14.

[4] Nieva-Echevarría B, Goicoechea E, Manzanos MJ, Guillén MD (2015). Usefulness of ^1^H NMR in assessing the extent of lipid digestion. Food Chem.

[5] Nieva-Echevarría B, Goicoechea E, Manzanos MJ, Guillén MD (2014). A method based on ^1^H NMR spectral data useful to evaluate the hydrolysis level in complex lipid mixtures. Food Res Int.

[6] Pivette P, Faivre V, Brubach JB, Daste G, Ollivon M, Lesieur S (2014). Polymorphism of glyceryl behenates: from the individual compounds to the pharmaceutical excipient. Chem Phys Lipids.

[7] Kushwah V, Lopes DG, Koutsamanis I, Plank H, Ardelean I, Sarkar A, Prpich A, am Ende MT, Schmidt HF, Doshi P, Shamblin SL, Laggner P, Paudel A (2020). Evolution of the microstructure and the drug release upon annealing the drug loaded lipid-surfactant microspheres. Eur J Pharm Sci.

[8] Brun M, Delample M, Harte E, Lecomte S, Leal-Calderon F (2014). Stabilization of air bubbles in oil by surfactant crystals: a route to produce air-in-oil foams and air-in-oil-in-water emulsions. Food Res Int.

[9] Fisher M, Kelly AP (1979). Multicenter trial of fluocinonide in an emollient cream base. Int J Dermatol.

[10] Moonen H, Bas H (2014). Mono- and diglycerides. Emulsifiers in food technology.

[11] Larsson K, Krog N (1973). Structural properties of the lipid-water gel phase. Chem Phys Lipids.

[12] Sein A, Verheij JA, Agterof WGM (2002). Rheological characterization, crystallization, and gelation behavior of monoglyceride gels. J Colloid Interface Sci.

[13] USP-NF 〈761〉 Nuclear Magnetic Resonance Spectroscopy. Available from: https://online.uspnf.com/uspnf/document/1_GUID-B980A615-B451-42FF-AC40-4CB8B64163E3_3_en-US?source=SearchResults&highlight=761. Accessed 2020 Oct 14.

[14] ICH topic Q 2 (R1) validation of analytical procedures: text and methodology step 5 note for guidance on validation of analytical procedures: text and methodology (CPMP/ICH/381/95) approval by CPMP November 1994 date for coming into operation. 1995.

[15] International Council for Harmonisation of Technical Requirements for Pharmaceuticals for Human Use Final Concept Paper ICH Q14: Analytical Procedure Development and Revision of Q2(R1) Analytical Validation.

[16] Gödecke T, Napolitano JG, Rodríguez-Brasco MF, Chen S-N, Jaki BU, Lankin DC, Pauli GF (2013). Validation of a generic quantitative ^1^H NMR method for natural products analysis. Phytochem Anal.

[17] Lachenmeier D, Schönberger T, Ehni S, Schütz B, Spraul M. A discussion about the potentials and pitfalls of quantitative nuclear magnetic resonance (qNMR) spectroscopy in food science and beyond. Proc XIII Int Conf Appl Magn Reson Food Sci. 2016;77. 10.1255/mrfs.15.

[18] Guideline for submitting samples and analytical data for methods validation. 1987.

[19] Guidance for industry Q2B validation of analytical procedures: Methodology. 1996.

[20] Yan KJ, Chu Y, Huang JH, Jiang MM, Li W, Wang YF, Huang HY, Qin YH, Ma XH, Zhou SP, Sun H, Wang W (2016). Qualitative and quantitative analyses of Compound Danshen extract based on ^1^H NMR method and its application for quality control. J Pharm Biomed Anal.

[21] Malz F, Jancke H (2005). Validation of quantitative NMR. J Pharm Biomed Anal.

[22] Nakka M, Nakka S (2018). Quantitative nuclear magnetic resonance spectroscopic method development and validation of sumatriptan succinate in pharmaceutical tablet dosage form. Int J Chem Tech Res.

[23] Parsons HM, Ekman DR, Collette TW, Viant MR (2009). Spectral relative standard deviation: a practical benchmark in metabolomics. Analyst..

[24] Monakhova YB, Diehl BWK (2020). A procedure for calibration transfer of DOSY NMR measurements: an example of molecular weight of heparin preparations. J Chemom.

[25] Schoenberger T. Guideline for qNMR analysis, DWG-NMR-001, ENFSI. 2019. Available from: http://enfsi.eu/wp-content/uploads/2017/06/qNMR-Guideline_version001.pdf. Accessed 2020 Oct 14.

[26] Diehl B, Holzgrabe U, Monakhova Y, Schönberger T (2020). Quo Vadis qNMR?. J Pharm Biomed Anal.

[27] Geisser J, Teipel J, Kuballa T, Weber S, Mildau G, Walch SG, Lachenmeier DW (2019). Requirements for accurate ^1^H NMR quantification of mineral oil hydrocarbons (paraffins) for pharmaceutical or cosmetic use. J Pharm Biomed Anal.

