# Supplementary material for: Quantitative Chemical Profiling of Commercial Glyceride Excipients via1H NMR Spectroscopy
Source: AAPS PharmSciTech. 2020 Dec 3;22(1):11. doi: 10.1208/s12249-020-01883-x (PMC7716940; doi:10.1208/s12249-020-01883-x)
Supplement: Supplementary file 1 — (DOCX 564 kb) [file 12249_2020_1883_MOESM1_ESM.docx]

**Quantitative chemical profiling of commercial glyceride excipients via ^1^H NMR spectroscopy**

Isha Saraf^1^, Varun Kushwah^1^, Hansjoerg Weber^2^, Dattatray Modhave^1^, Thean Yeoh^3^, and Amrit Paudel^1, 4*^

^1^Research Center Pharmaceutical Engineering (RCPE) GmbH, Inffeldgasse 13, 8010 Graz, Austria

^2^Institute of Organic Chemistry, Graz University of Technology, Stremayrgasse 9A, 8010 Graz

Austria

^3^Drug Product Design, Pharmatherapeutics Pharmaceutical Sciences, Worldwide Research and Development, Pfizer Inc, Groton, CT, USA

^4^Institute for Process and Particle Engineering, Graz University of Technology, Inffeldgasse 13, 8010 Graz, Austria

^*^Corresponding author:

E-mail: [amrit.paudel@tugraz.at](mailto:amrit.paudel@tugraz.at)

Tel.: +43 316 873 30912

**Supplementary Information**

**T1 relaxation experiment:**

T1-Relaxation experiments were performed using the standard mixture (glycerol, mono-, di-glycerides and their isomers, tri-glycerides and fatty acids) dissolved in 0.6 mL of CDCl_3_ containing 1.0 mg of reference standard (tCNB) as per the protocol mentioned in the section 3.1.

T1-Relaxation experiments have been carried out using the inversion-recovery sequence (d1 - 180° pulse - d2 - 90° pulse - acq) and the following parameters: d1 20 s, number of scans 4, d2 as an array of 10 values from 0.0625 - 32 s.

**Result and Discussion**

The T1 relaxation values obtained were less than 3 secs for all the signals (Figure S1, S2 and S3). Whereas, for the selected signals used for the quantification of chemical composition (equation 2 to 8) of MDGs, the T1 relaxation values were even lower than 2 sec, suggesting application of sufficient relaxation times for the proton (relaxation delay 1.0 s, number of scans 32, acquisition time 2.049 s).


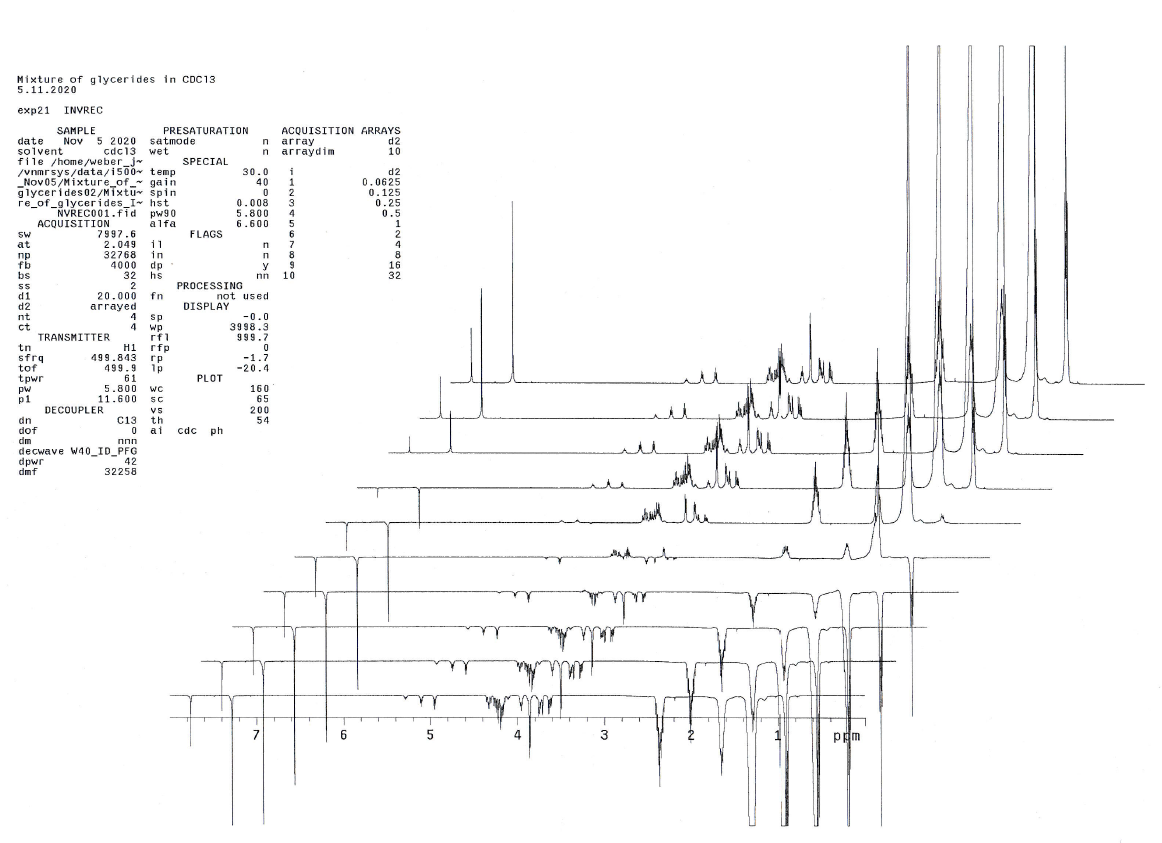


Figure S1: T1 Relaxation experiments using the inversion-recovery sequence


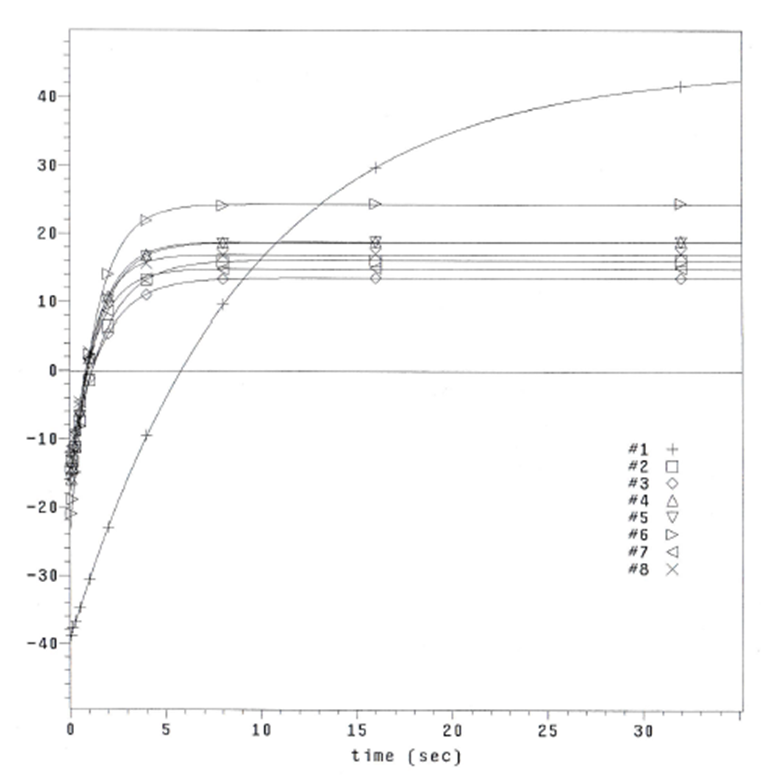


Figure S2: Relaxation curves of samples (8 most intense peak from the Figure S1)


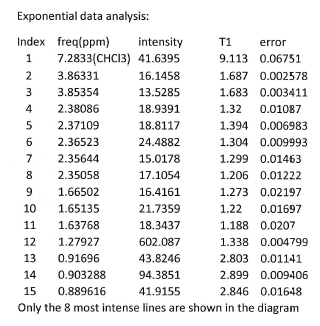


Figure S3: T1 relaxation time value (of 15 most intense signals from Figure S1)
